# *CCL3L* Copy Number Variation and the Co-Evolution of Primate and Viral Genomes

**DOI:** 10.1371/journal.pgen.1000359

**Published:** 2009-01-30

**Authors:** German Gornalusse, Srinivas Mummidi, Weijing He, Guido Silvestri, Mike Bamshad, Sunil K. Ahuja

**Affiliations:** 1 Veterans Administration Research Center for AIDS and HIV-1 Infection, South Texas Veterans Health Care System, and Department of Medicine, University of Texas Health Science Center, San Antonio, Texas, United States of America; 2 Department of Microbiology/Immunology, University of Texas Health Science Center, San Antonio, Texas, United States of America; 3 Departments of Pathology and Laboratory Medicine, University of Pennsylvania School of Medicine, Philadelphia, Pennsylvania, United States of America; 4 Departments of Pediatrics and Genome Sciences, University of Washington and Seattle Children's Hospital, Seattle, Washington, United States of America; 5 Department of Biochemistry, University of Texas Health Science Center, San Antonio, Texas, United States of America; Fred Hutchinson Cancer Research Center, United States of America

“Let it … be borne in mind how infinitely complex and close-fitting are the mutual relations of all organic beings to each other and to their physical conditions of life; and consequently what infinitely varied diversities of structure might be of use to each being under changing conditions of life.” — Charles Darwin, On the Origin of Species

November 24, 2008, marked the 149th anniversary of the first publication of Charles Darwin's seminal work entitled “On the Origin of Species.” The above quote comes from Darwin's answer to his question about how the struggle for existence might shape patterns of variation. Of course, in the 19th century, Darwin was making inferences simply based on observations of morphological variation. Yet, if he were alive today, he would be struck by how prescient his statement was, even as applied to questions about the long co-evolution of primates and viral pathogens, including lentiviruses [1].

The human genome was originally thought to be structurally stable, but it turns out to be quite dynamic, with many genomic regions duplicated or deleted among individuals to the extent that they exist in variable copy numbers. Within these copy number variations (CNVs), genes that encode proteins involved in immune responses are over-represented [2], including chemokines that play key roles in host defense against infectious diseases [3]–[5]. This observation implies that our genomes have DNA sequences that may memorialize immune strategies used to combat ancient pathogens. The past 5 years have witnessed an intense interest in understanding the extent of CNV in primate genomes [6],[7] and their contributions to disease susceptibility in humans [8]. In this issue of *PLoS Genetics*, Degenhardt and colleagues provide a link between CNV and disease susceptibility in non-human primates [9].

Asian macaques—including rhesus, pigtail, and cynomolgus—are commonly used as animal models to study the determinants of AIDS pathogenesis and evaluate HIV-1 vaccine candidates. After being challenged with Simian Immunodeficiency Virus (SIV)—the simian counterpart of HIV—some macaques rapidly develop features similar to AIDS, whereas others do so more slowly. A similar clinical conundrum exists in humans, as many people who are HIV-1–positive progress rapidly to AIDS, whereas others resist disease progression, despite not receiving antiretroviral therapy. Both viral and host factors contribute to the variability in AIDS progression rates in humans [10].

Among host factors that may contribute to variable HIV-AIDS susceptibility, significant attention has focused on the role of variations in genes that influence HIV transmission, such as the genes that encode CC chemokine receptor 5 (CCR5), the major HIV co-receptor required for cell entry of virus, and CCR5 chemokine ligands such as CC ligand 3 (CCL3) and its paralog CCL3L1 [10]. For example, homozygosity for a 32-bp deletion in the coding sequence of *CCR5* abolishes CCR5 expression and confers near-absolute protection against acquiring HIV [10]. CCR5 ligands can block entry of HIV into cells by “gumming” up the site on CCR5 to which HIV-1 binds and by reducing cell surface expression of CCR5 [5]. Among the chemokines that bind to CCR5, CCL3L1 has the most potent HIV-suppressive properties [5]. Additionally, *CCL3L* genes were shown to be subject to CNV in humans and chimpanzee [11],[12]. A low copy number of the *CCL3L1*-containing segmental duplication was found to be associated with reduced CCL3/CCL3L1 chemokine levels, reduced chemotaxis of CCR5-expressing cells, and reduced proportions of HIV target cells that express CCR5 [5],[11],[12]. This discovery prompted investigators to inquire whether intersubject differences in *CCL3L1* copy number might be a basis for variable HIV-AIDS susceptibility. A low copy number of the *CCL3L1*-containing segmental duplication was shown to be associated with or correlate with an increased risk of acquiring HIV infection [12]–[17], a faster rate of progression to AIDS or CD4+ T cell depletion [12],[16],[18],[19], higher HIV viral loads [12],[13],[20], lower HIV-specific immune responses [20], and lower cell-mediated immune responses [18].

In this issue of *PLoS Genetics*, Degenhardt and colleagues tested whether a low *CCL3L* copy number was associated with a faster rate of progression to AIDS in macaques challenged experimentally with SIV [9]. They found that macaques with a low copy number of *CCL3L* genes experience a significantly more rapid rate of progression to experimental AIDS, with the *CCL3L* CNV accounting for ∼18% of the variability in experimental AIDS progression rates.

Indian rhesus macaques progress more quickly to experimental AIDS than do Chinese macaques [21]. Degenhardt et al. suggest that the lower *CCL3L* copy number in Indian rhesus macaques may underlie the more rapid progression to AIDS in Indian versus Chinese macaques. Thus, in addition to serving as a determinant of interindividual differences in the outcome of experimental AIDS, *CCL3L* gene dose may account for some of the observed interpopulation differences in simian AIDS progression rates.

Previous studies have shown that there is a clear genetic distinction between rhesus macaques that originate from India versus China [22]. Thus, population structure is a possible confounding variable whenever phenotypic differences between these populations are investigated. Degenhardt et al. controlled for population structure using a battery of microsatellites and demonstrated that the *CCL3L* CNV was a better predictor of outcome than population affiliation. While other genes may also influence progression to simian AIDS in experimentally infected rhesus macaques [23], Degenhardt et al.'s results show that the *CCL3L* CNV has strong effects on progression to simian AIDS. These results have practical implications for efforts to develop an effective HIV vaccine. To distinguish more clearly between vaccine efficacy and intrinsic variation in host response, it may be important to stratify rhesus macaques by *CCL3L* CNV.

Understanding the role of chemokine CNVs in primate disease is made more complicated by several observations. At least in humans, there are multiple *CCL3L* (*CCL3L1*, *CCL3L2*, and *CCL3L3*) and *CCL4L* (*CCL4L1* and *CCL4L2*, paralogs of *CCL4*) genes, which are found on chromosome 17q12; a similar diversity might exist in nonhuman primates (Figure 1A). However, the human *CCL3L-CCL4L–*containing locus has been subjected to complex homologous recombination events [24],[25], such that individuals may vary not only in the total copy number of *CCL3L* and *CCL4L* genes but also their individual components [11],[16]. Furthermore, the mRNA structure of the different *CCL3L* and *CCL4L* genes appears to vary (Figure 1B and 1C). For example, while human *CCL4L1* and *CCL4L2* share 100% sequence identity in the coding regions, a fixed mutation at the intron–exon boundary of *CCL4L1* results in the production of aberrantly spliced transcripts (Figure 1B and 1C), and a higher *CCL4L1* copy number has been associated with an increased risk of acquiring HIV infection [26] and faster rate of progression to AIDS [16]. With these features in mind, future studies will need to consider such questions as: Are the different copies of *CCL3L* and *CCL4L* in rhesus macaques identical or do they encode transcripts/proteins with different functions? How many of these copies are actually pseudogenes? Similar to what is observed in humans [16],[26], could *CCL4L* genes also contribute to simian AIDS independently of or in combination with distinct *CCL3L* genes? Could such complexity also confound genotype–phenotype studies in humans that investigate the relationship between *CCL3L* or *CCL4L* CNV with disease susceptibility? In addition to these *CCL3L-CCL4L–*related genetic factors that may complicate the analyses of association studies, there might be other confounders to consider. For example, co-infection with other viruses (e.g., hepatitis C virus [HCV]) may modify the association between *CCL3L1* copy number and risk of acquiring HIV infection [17].

**Figure 1:**
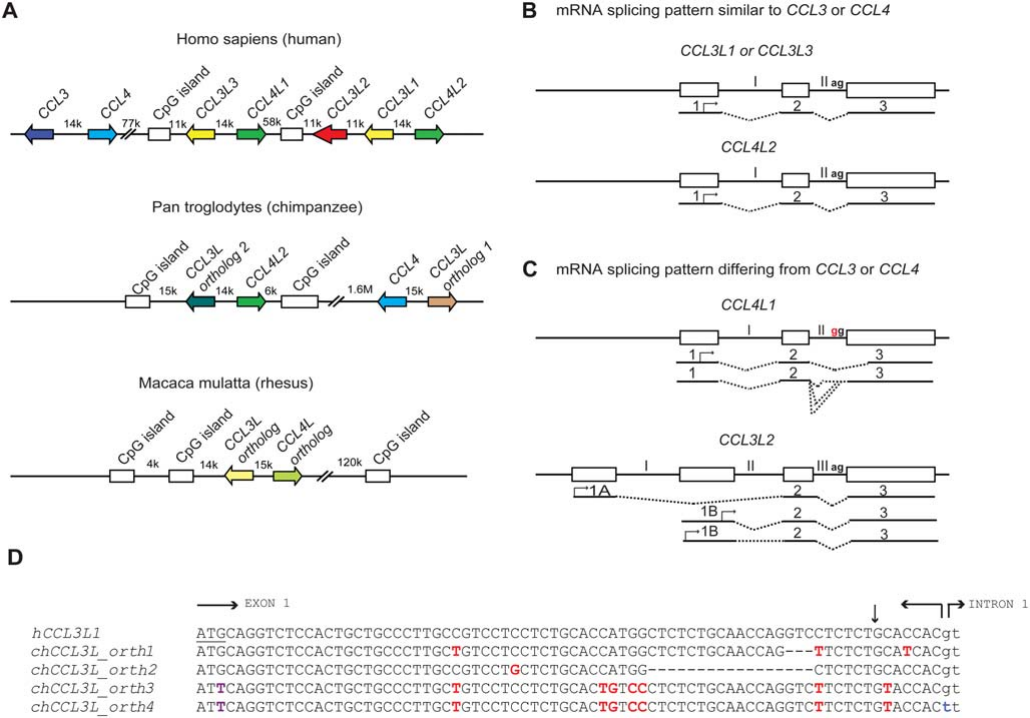
Comparative genomics of primate CCL3L and CCL4L loci. (A) Comparison of *CCL3L* and *CCL4L* in human and nonhuman primates. The top panel shows a schema of the chemokine locus at human chromosome 17q12 based on the NT_010799.14 contig. *CCL3* and *CCL4* exist as single-copy genes per haploid genome. The genes encoding the non-allelic isoforms of *CCL3* (National Center for Biotechnology Information gene ID given in parentheses) are denoted as *CCL3L1* (6349), *CCL3L2* (390788), and *CCL3L3* (414062) and those of *CCL4* are denoted as *CCL4L1* (9560) and *CCL4L2* (388372). The middle panel shows a schema of the *CCL3L* and *CCL4L* locus in chimpanzee based on the chromosome 17NW_001226927.1 contig. *CCL3L* orthologs (denoted as “1” and “2”) map ∼ 1.6 Mb apart in this contig. In contrast to the human locus, chimpanzee contigs lack *CCL3L2*. The bottom panel shows a schema of the *CCL3L* and *CCL4L* locus in rhesus monkey based on chromosome 16 NW_001103987 contig. Of note, other orthologs of *CCL3L* and *CCL4L* were found in two other rhesus contigs (NW_001103644.1 and NW_001102959). CpG islands found in primate *CCL3L* and *CCL4L* loci are also depicted. Distances between genes are approximate, and the map is not to scale. The arrows denote the orientation of the genes. k, kb; M, Mb. (B and C) Schematic representation of genomic and mRNA structure of human *CCL3L* and *CCL4L* genes that have mRNA splicing patterns that are similar (B) or dissimilar (C) to *CCL3* and *CCL4.* Exons are represented as boxes and introns as connecting lines labeled with Roman numbers; the splicing pattern is denoted by the dashed lines. *CCL3L1*, *CCL3L3*, and *CCL4L2* are each composed of three exons, and the start codon (denoted with an arrow) is located in the first exon. *CCL4L1* has a transition in the splicing acceptor site located in intron II (AG→GG, indicated in red), which results in the generation of aberrantly spliced transcripts that use alternative acceptor sites located either in the intron II or in the third exon [26]. *CCL3L2* was previously considered as a pseudogene [5]. However, recent studies in our lab suggest that it has a four exon structure and is predicted to transcribe alternatively spliced mRNA species with open reading frames (ORFs) that contain chemokine-like domains [16]; CCL3L2 mRNA transcripts originate from two novel upstream exons (designated as 1A and 1B) and are linked to the second and third exons, which are homologous to exons 2 and 3 found in *CCL3L1* or *CCL3L3*. (D) Nucleotide sequence of human *CCL3L1* (or *CCL3L3*) and its alignment with four distinct chimpanzee *CCL3L* (*chCCL3L*) orthologous genes from the translation initiation site until the start of intron 1. The translational start codon in *hCCL3L1* is underlined. Horizontal arrows delimit the exon–intron boundaries. Dashes indicate deletions. Polymorphic sites relative to the *hCCL3L1* are shown in red. The vertical arrow represents the site for signal peptidase cleavage. *chCCL3L ortholog 1* is predicted to encode a chemokine with amino acids that are shared with both hCCL3L1 and hCCL3. *chCCL3L ortholog 2* has a deletion of 17 nucleotides (relative to *hCCL3L1*) that may lead to loss of the signal peptide cleavage motif. Notably, two additional and different *CCL3L* orthologs were found in two independent chimpanzee contigs, denoted as NW_001227489.1 (ortholog 3) and NW_001227474.1 (ortholog 4), which have a mutation at the translation initiation site (shown in purple) and differ from each other in the splicing donor site of intron 1 (shown in blue) and other genomic regions (unpublished data). Of note, all four *chCCL3L1* orthologs had sequences that were completely homologous to the primer–probe sets used to detect *CCL3L* CNV in humans and chimpanzee previously [12] and by Degenhardt et al. [9]. All the chimpanzee orthologs are also predicted to encode transcripts with potential ORFs with chemokine-like domains. The accession numbers for the predicted ORFs encoded by chimpanzee *CCL3L* orthologs 1, 2, 3, and 4 are NP_001029254, XP_001152451, XP_001172388, and XP_001172226, respectively.

Using real-time PCR-based approaches, Degenhardt et al. confirmed an earlier report that chimpanzees have variable copy numbers of *CCL3L* genes [9],[12], as do other nonhuman primates including orangutan, African green monkey, and Sooty Mangabey; on average the *CCL3L* copy numbers in nonhuman primates are much higher than those found in human populations [9],[12]. Furthermore, analyses of the chimpanzee genome (from the Clint reference sequence) revealed at least four distinct *CCL3L* genes (Figure 1D). These results differ with those of Perry et al., who, using an array-based method, found that chimpanzees have two *CCL3L* copies per diploid genome [7]. These contrasting results underscore the challenges of accurately quantifying CNVs, a particularly important issue given the intense interest in understanding the role of CNVs in disease susceptibility [8].

One possible reason for the extensive variability in *CCL3L* copy number in primates may reflect that the variability represents an ancient host defense mechanism. While this hypothesis needs to be tested with additional empirical data, it is consistent with the observation that there is a parallel to primate chemokine CNV in viruses: many viral pathogens have hijacked DNA sequences found in primates and adapted them to encode chemokine receptors and chemokines that specifically target and, in some cases, neutralize the primate chemokine system [27]. These viral-encoded antichemokine strategies highlight the importance of the chemokine system in host defense against infections.

Darwin, an astute observer of nature, might ask, “Why does there appear to be so much structural variation for genes encoding chemokines?” The ancient and dynamic battle between mammalian hosts and pathogens has exerted unrelenting selection pressure on the host genome, promoting the development of a complex and adaptable immune system. Conversely, the successful replication and persistence of latent viruses within the mammalian host implies that they have evolved the means to evade or manipulate host immune defenses. In the case of viruses, it is clear that they have targeted the immune responses mediated by chemokines [27]. Is the expansion and diversification of the chemokine gene family, as a consequence of gene duplication [3],[4], evidence of the co-evolution of host defenses and viral pathogens? The elegant study by Degenhardt et al. gets us closer to answering this question, but much work remains. Nevertheless, Darwin would be pleased that the paradigm he established more than a century ago continues to be robust for explaining the “varied diversities of structure.”

## References

[1] Gifford RJ, Katzourakis A, Tristem M, Pybus OG, Winters M (2008). A transitional endogenous lentivirus from the genome of a basal primate and implications for lentivirus evolution.. Proc Natl Acad Sci U S A..

[2] Bailey JA, Gu Z, Clark RA, Reinert K, Samonte RV (2002). Recent segmental duplications in the human genome.. Science.

[3] Rot A, von Andrian UH (2004). Chemokines in innate and adaptive host defense: basic chemokinese grammar for immune cells.. Annu Rev Immunol.

[4] Zlotnik A, Yoshie O, Nomiyama H (2006). The chemokine and chemokine receptor superfamilies and their molecular evolution.. Genome Biol.

[5] Menten P, Wuyts A, Van Damme J (2002). Macrophage inflammatory protein-1.. Cytokine Growth Factor Rev.

[6] Redon R, Ishikawa S, Fitch KR, Feuk L, Perry GH (2006). Global variation in copy number in the human genome.. Nature.

[7] Perry GH, Yang F, Marques-Bonet T, Murphy C, Fitzgerald T (2008). Copy number variation and evolution in humans and chimpanzees.. Genome Res.

[8] Couzin J (2008). Human genetics. Interest rises in DNA copy number variations–along with questions.. Science.

[9] Degenhardt JD, de Candia P, Chabot A, Schwartz S, Henderson L (2009). Copy number variation of *CCL3*-like genes affects rate of progression to simian-AIDS in rhesus macaques (*Macaca mulatta*).. PLoS Genet.

[10] Telenti A, Carrington M (2008). Host factors associated with outcome from primary human immunodeficiency virus-1 infection.. Curr Opin HIV AIDS.

[11] Townson JR, Barcellos LF, Nibbs RJ (2002). Gene copy number regulates the production of the human chemokine CCL3-L1.. Eur J Immunol.

[12] Gonzalez E, Kulkarni H, Bolivar H, Mangano A, Sanchez R (2005). The influence of CCL3L1 gene-containing segmental duplications on HIV-1/AIDS susceptibility.. Science.

[13] Kuhn L, Schramm DB, Donninger S, Meddows-Taylor S, Coovadia AH (2007). African infants' CCL3 gene copies influence perinatal HIV transmission in the absence of maternal nevirapine.. AIDS.

[14] Meddows-Taylor S, Donninger SL, Paximadis M, Schramm DB, Anthony FS (2006). Reduced ability of newborns to produce CCL3 is associated with increased susceptibility to perinatal human immunodeficiency virus 1 transmission.. J Gen Virol.

[15] Nakajima T, Ohtani H, Naruse T, Shibata H, Mimaya JI (2007). Copy number variations of CCL3L1 and long-term prognosis of HIV-1 infection in asymptomatic HIV-infected Japanese with hemophilia.. Immunogenetics.

[16] Shostakovich-Koretskaya L, Catano G, Chykarenko ZA, He W, Gornalusse G (2009). Combinatorial content of CCL3L and CCL4L gene copy numbers influence HIV-AIDS susceptibility in Ukrainian children.. AIDS. In Press.

[17] Sadam M, Karki T, Huik K, Avi R, Rüütel K (2008). CCL3L1 Variable gene copy number influence on the susceptibility to HIV-1/AIDS among Estonian intravenous drug users..

[18] Dolan MJ, Kulkarni H, Camargo JF, He W, Smith A (2007). CCL3L1 and CCR5 influence cell-mediated immunity and affect HIV-AIDS pathogenesis via viral entry-independent mechanisms.. Nat Immunol.

[19] Ahuja SK, Kulkarni H, Catano G, Agan BK, Camargo JF (2008). CCL3L1-CCR5 genotype influences durability of immune recovery during antiretroviral therapy of HIV-1-infected individuals.. Nat Med.

[20] Shalekoff S, Meddows-Taylor S, Schramm DB, Donninger SL, Gray GE (2008). Host CCL3L1 gene copy number in relation to HIV-1-specific CD4+ and CD8+ T-cell responses and viral load in South African women.. J Acquir Immune Defic Syndr.

[21] Ling B, Veazey RS, Luckay A, Penedo C, Xu K (2002). SIV(mac) pathogenesis in rhesus macaques of Chinese and Indian origin compared with primary HIV infections in humans.. AIDS.

[22] Hernandez RD, Hubisz MJ, Wheeler DA, Smith DG, Ferguson B (2007). Demographic histories and patterns of linkage disequilibrium in Chinese and Indian rhesus macaques.. Science.

[23] Giraldo-Vela JP, Rudersdorf R, Chung C, Qi Y, Wallace LT (2008). The major histocompatibility complex class II alleles Mamu-DRB1*1003 and -DRB1*0306 are enriched in a cohort of simian immunodeficiency virus-infected rhesus macaque elite controllers.. J Virol.

[24] Perry GH, Ben-Dor A, Tsalenko A, Sampas N, Rodriguez-Revenga L (2008). The fine-scale and complex architecture of human copy-number variation.. Am J Hum Genet.

[25] Cardone MF, Jiang Z, D'Addabbo P, Archidiacono N, Rocchi M (2008). Hominoid chromosomal rearrangements on 17q map to complex regions of segmental duplication.. Genome Biol.

[26] Colobran R, Adreani P, Ashhab Y, Llano A, Este JA (2005). Multiple products derived from two CCL4 loci: high incidence of a new polymorphism in HIV+ patients.. J Immunol.

[27] Boomker JM, de Leij LF, The TH, Harmsen MC (2005). Viral chemokine-modulatory proteins: tools and targets.. Cytokine Growth Factor Rev.

